# Social inequities in the community food environment: temporal analysis of food retail availability in the state of Rio Grande do Sul, Brazil (2010–2022)

**DOI:** 10.1017/S1368980025101286

**Published:** 2025-10-14

**Authors:** Dafne Pavão Schattschneider, Elma Izze da Silva Magalhães, Ariene Silva do Carmo, Lauren Yurgel da Silva, Carolina Machado Colucci, Júlio Celso Borello Vargas, Raquel Canuto

**Affiliations:** 1 Postgraduate Program in Food, Nutrition and Health, Universidade Federal do Rio Grande do Sul, Room 220, 2nd floor, 2400 Ramiro Barcelos Street, Floresta, Porto Alegre, RS 90035-003, Brazil; 2 Postgraduate Program in Food, Nutrition and Health, Universidade do Estado do Rio de Janeiro, Room 12023, 12th floor, Block D, 524 São Francisco Xavier Street, Maracanã, Rio de Janeiro 20550-013, Brazil; 3 Faculty of Nutrition, Universidade Federal do Rio Grande do Sul, 2400 Ramiro Barcelos Street, Floresta, Porto Alegre, RS 90035-003, Brazil; 4 Postgraduate Program in Urban and Regional Planning, Universidade Federal do Rio Grande do Sul, 320 Sarmento Leite Street, 5th floor, Farroupilha, Porto Alegre, RS 90050-170, Brazil

**Keywords:** Food environment, Social determinants of health, Socio-economic disparities in health, Ecological study

## Abstract

**Objective:**

To describe changes in the community food environment between 2010 and 2022 in all municipalities in the state of Rio Grande do Sul (RS), Brazil, and to evaluate the possible associated sociodemographic inequities.

**Design:**

This ecological study was based on an analysis of the distribution and density of food retail establishments between 2010 and 2022 and their associations with the sociodemographic characteristics of the municipalities. Sociodemographic and food retail variables were extracted from secondary government databases. The establishments were classified according to the degree of processing of the foods they predominantly sold. Non-parametric tests and linear and Prais–Winsten regressions were used to analyse data.

**Setting:**

State of RS, Brazil.

**Participants:**

All 497 municipalities.

**Results:**

There was a significant reduction in overall food retail density (Coef.: –2·97; 95 % CI: –3·34, –2·61; *P* < 0·001). The greatest reduction occurred in establishments that sourced ultra-processed foods (Coef.: –3·34; 95 % CI: –3·65, –3·02; *P* < 0·001), which, despite the decrease, remained the most widely present. In 2022, the density of these establishments (median: 24·5; min/max: 4·4–124·8) was twice the density of establishments supplying fresh/minimally processed foods and culinary ingredients (median: 13·1; min/max: 0·0–95·8). Cities with greater social vulnerability had lower densities of establishments and greater reductions in the density of establishments over the evaluated period.

**Conclusions:**

The reduction in food retail outlets disproportionately affected the most vulnerable municipalities and negatively impacted the availability of healthy foods. These findings reinforce the need for food and nutrition policies that promote equity in the food environment.

Food systems can be conceptualised as a set of elements and related processes from the production to the consumption and final disposal of food that encompass the effects that these processes can have on health, society and the environment^(1)^. The food environment is the interface between the acquisition of food by people within the food system^(1)^ and the political and sociocultural aspects that affect them^(2)^. Glanz *et al*. proposed the first conceptual model for the study of food environments and classified them into four subdivisions^(3)^; among them, the community food environment encompasses aspects such as the type, location and opening hours of food establishments and how individuals access these spaces. Studying the community food environment is important for understanding how the availability of and access to food differ among populations and can reveal social inequalities that influence food consumption, social and environmental dynamics and the health and well-being of communities^(1–3)^.

In recent decades, changes have been observed in the community food environment, food supply and, consequently, food consumption^(1,4–6)^. These changes are marked by an increase in access to ultra-processed foods, which favours weight gain and an increase in chronic noncommunicable diseases, such as diabetes mellitus^(4,5,7)^.

The community food environment is dynamic and can change over short periods of time^(8)^. Most studies that have evaluated changes in the community food environment have observed an increase in all types of establishments^(8–13)^, especially those with a predominant sale of unhealthy foods^(9,11,14,15)^. However, studies conducted in the Global North have pointed to factors that contributed to the closure of establishments, such as the COVID-19 pandemic^(16,17)^ and economic instability in socially and racially disadvantaged communities^(7,18)^. In Brazil, the two studies found that assessed changes in the community food environment also identified social inequities. Nevertheless, differently from studies in the Global North, these inequities were marked by a lower density of establishments with healthy food sales patterns over the years in lower-income census tracts^(11)^ and by a reduction in supermarkets in territories with higher social vulnerability^(19)^.

Investigating changes in the community food environment over time is important both for monitoring the availability of food and for understanding the process of nutritional transition in territories. In addition, identifying possible patterns of social disparities in food availability is essential for the development of public policies on food and nutrition and food and nutrition security, with a focus on mitigating social inequities in health^(1,4,6)^. However, most studies on the subject have been conducted in the Global North^(8,9,13,15,18,20–23)^, and only two studies have been conducted in Brazil, both in the municipality of Belo Horizonte, in southeastern Brazil^(11,19)^. Therefore, the objective of this study is to describe changes in the community food environment between 2010 and 2022 in all municipalities in the state of Rio Grande do Sul, Brazil, and to evaluate the potential sociodemographic inequalities associated with these changes.

## Methodology

### Design, location and sample of the study

This is an ecological study whose units of analysis are the municipalities in the state of Rio Grande do Sul, Brazil, in the years 2010 and 2022. Rio Grande do Sul is the southernmost state of Brazil and has 10 882 965 inhabitants. Its Human Development Index is 0·771 (high), and it ranks the 5th highest in the country among the twenty-six states plus the Federal District^(24)^. Within the state, 23·3 % of individuals (2 340 378) belong to Black, Brown or Indigenous ethnic minorities, according to the 2022 Demographic Census^(25)^.

The study included 497 municipalities in the state in 2022 and 496 municipalities in 2010, as a municipality was created during this period.

### Assessment of the community food environment

The community food environment was assessed according to the availability of retail food. Food retail data from all municipalities from 2010 to 2022 were extracted from the Annual Report of Social Information (RAIS), a government database officially used for describing the food environment by the Brazilian government^(26)^ and in academic studies^(27,28)^. This database provides data such as the type of establishment and its location on the basis of the postal address code (CEP) of each establishment in the country. The establishments are classified by the National Registry of Economic Activity (CNAE), which is officially adopted by the National Statistical System. The Secretary of Labour of the Brazilian Ministry of Economy requests information from legal entities and other employers annually to standardise the classification of companies according to their main economic activities, which allows the identification of the type of establishment (e.g. supermarket, fruit and vegetable market, canteen).

On the basis of the RAIS data, the food outlets were classified according to the *Locais-Nova* methodology for the southern region of Brazil, which was proposed by Silva *et al*.^(29)^. *Locais-Nova* is based on the NOVA classification, which is a widely recognised system that categorises foods according to the extent and purpose of their processing. It includes four groups: (G1) unprocessed or minimally processed foods, such as fruits, vegetables, grains, milk and eggs, which preserve their natural characteristics; (G2) processed culinary ingredients like oils, sugar and salt, used in cooking; (G3) processed foods, such as canned vegetables and cheeses, made by adding culinary ingredients to whole foods; and (G4) ultra-processed foods, which are industrial products made mostly from food-derived substances and additives, such as soft drinks, packaged snacks and instant meals^(30)^.

The *Locais-Nova* methodology used data from the 2017–2018 Brazilian Household Budget Survey to categorise types of establishments according to household food acquisition profiles. For this categorisation, cut-off points were established on the basis of the average percentage contribution of each food group to the total grams purchased in the country and in each region of the country. The average percentage of the contribution of each food group at each place of acquisition was compared. When the average percentage contribution of food groups in a given place of acquisition was equal to or greater than the average contribution of the country or region under analysis, that place was classified as a source of acquisition for that food group. Unprocessed or minimally processed foods and processed culinary ingredients were evaluated as a single group (G1 + G2) in the *Locais-Nova* methodology^(29)^. The final classification of food retail establishments into groups G1 + G2, G3 and G4, for the present study, is presented in Table 1.

**Table 1:** Food establishments according to the *Locais-Nova* classification

G1 + G2: Fresh/minimally processed foods and culinary ingredients
Butcher shops
Fish markets
Fruit and vegetable markets
Canteens
Restaurants
Street vendors
Supermarkets
Hypermarkets
G3: Processed foods
Bakery and pastry product stores
Bakeries
Dairy and cold cuts stores
G4: Ultra-processed foods
Bars
Retail of beverages not consumed on the premises
Convenience stores
Candy and confectionery stores
Snack bars
Small grocery stores
General or unspecified food product stores
Prepared food outlets
Bakery and pastry product stores
Bakeries
Supermarkets
Hypermarkets

Subsequently, to characterise the food retail, the total number and density (per 10 000 inhabitants) of each type of food retail outlet per municipality were calculated. Density was calculated by dividing the total number of establishments by the population of the municipality, and the result was multiplied by 10 000.

### Sociodemographic characteristics of the municipalities

Secondary publicly accessible data for the years 2010 and 2022 were analysed. Sociodemographic variables were obtained from the United Nations Development Programme^(31)^ (Municipal Human Development Index – MHDI) and from the Demographic Censuses of the Brazilian Institute of Geography and Statistics (IBGE) in 2010 (which included population, race/colour, illiteracy rate, average household income) and 2022 (which included population, race/colour, illiteracy rate)^(25)^. In 2022, only the variables provided by the 2022 Census up to the time of analysis were used. For the variable municipality size, municipalities were categorised according to the classification proposed by the Brazilian government in previous studies^(26)^ as very small 1 (≤ 20 000 inhabitants), small 2 (> 20 000 to ≤ 50 000 inhabitants), medium (> 50 000 to ≤ 100 000 inhabitants) or large/metropolis (> 100 000 inhabitants). The percentage of ethnic-racial minorities (self-reported as Black, Brown or Indigenous) was categorised using the 50th percentile of the variable distribution as a reference. Thus, this indicator was ≤ 15 % or > 15 % of ethnic-racial minorities in 2010 and ≤ 20 % or > 20 % of ethnic-racial minorities in 2022. The illiteracy rate was also categorised using the 50th percentile of the variable distribution as a reference, with ≤ 7 % or > 7 % in 2010 and ≤ 5 % or > 5 % in 2022. The MHDI was classified as very low, low, medium or high. For the present study, the MHDI variable was categorised into the categories of low/medium (from 0·500 to 0·6999) and high/very high (from 0·700 to 1) since no municipality was classified as having very low MHDI, only one municipality had low MHDI and only one municipality had very high MHDI. The mean household income was categorised into tertiles (1st tertile: 325·47–616·1; 2nd tertile: 616·28–808·17; 3rd tertile: 811·05–1722·37).

### Data analysis

Data were analysed using Stata software, version 18.0. The variables were tested for normality using the Shapiro–Wilk test. Continuous variables were described as medians, interquartile ranges, means and sd, whereas categorical variables were described as absolute and relative frequencies. The medians of the absolute number and density of food establishments in 2010 and 2022 were compared using the Wilcoxon test.

Mann–Whitney or Kruskal–Wallis tests were used to compare the medians of the types of food outlets in relation to the sociodemographic variables of the municipalities. Prais–Winsten regression was used to evaluate the temporal trend in food retail in RS from 2010 to 2022. The analyses were conducted using annual state-level aggregated data (i.e. one observation per year). To explore potential social inequities, we stratified the analyses according to the size of the municipality, mean family income, MHDI, race and illiteracy rate, using data from the 2010 census to categorise the municipalities according to sociodemographic characteristics. In each stratum, a separate state-level time series was analysed. Simple linear regression was used to evaluate changes in food retail availability in the state before and after the COVID-19 pandemic (between 2020 and 2022). In all analyses, a significance level of 5 % was considered. In addition, maps were constructed to characterise the community food environment of RS during the years analysed with the aid of QGIS software version 34.9.

## Results

Regarding the sociodemographic variables evaluated, among the 496 municipalities analysed in 2010, 64·5 % had high/very high MHDI, and the median household income was Brazilian Real (BRL) 701·59 (min: BRL 325·47; max: BRL 1722·37), equivalent to 137·56 % of the minimum wage that year (BRL 510·00). The mean of the percentages of ethnic-racial minorities and illiteracy rates of the municipalities was 14·5 % (sd: 7·83) and 6·6 % (sd: 3·33), respectively. In 2022, among the 497 municipalities analysed, the mean of the percentages of ethnic-racial minorities was 18·6 % (sd: 8·29), and the mean of the illiteracy rates was 4·7 % (sd: 2·39).

Of the 497 municipalities analysed, a reduction was observed for all food retail groups between 2010 and 2022, with similar reductions across establishments that source fresh/minimally processed foods and culinary ingredients, those that source processed foods and those that source ultra-processed foods. The only exception was supermarkets, which presented no significant changes over the period, as shown in Table 2. However, in the Prais-Winsten regression used to evaluate the time trend of all years from 2010 to 2022, supermarkets showed a small reduction over the period (Coef: –0·02; 95 % CI: –0·63, –0·34; *P* = 0·009; online Supplementary Table 2).

**Table 2:** Total number of food retail establishments by type in the municipalities of Rio Grande do Sul, Brazil, in 2010 and 2022

Type of establishment	2010				2022				*P*-value ^*^
	Mean	sd	Median	Min.–max.	Mean	sd	Median	Min.–max.	
Fresh/minimally processed foods and culinary ingredients	49·5	191·9	12·0	0·0–3827·0	34·9	151·9	8·0	0·0–95·8	< 0·0001
Butcher shops	7·1	19·1	2·0	0·0–265·0	2·8	9·5	0·0	0·0–142·0	< 0·0001
Fish markets	0·5	2·1	0·0	0·0–34·0	0·3	1·1	0·0	0·0–17·0	< 0·0001
Fruit and vegetable markets	3·8	10·9	1·0	0·0–135·0	2·2	6·3	0·0	0·0–89·0	< 0·0001
Canteens	0·4	1·9	0·0	0·0–30·0	0·2	1·5	0·0	0·0–29·0	< 0·0001
Restaurants	24·5	129·5	5·0	0·0–2726·0	21·1	117·9	3·0	0·0–2431·0	< 0·0001
Street vendors	1·6	6·1	0·0	0·0–107·0	0·7	2·4	0·0	0·0–38·0	< 0·0001
Supermarkets	7·6	17·2	3·0	0·0–268·0	7·0	15·4	3·0	0·0–240·0	0·0678
Hypermarkets	3·9	15·1	1·0	0·0–276·0	0·5	2·8	0·0	0·0–55·0	< 0·0001
Processed foods	12·4	40·7	3·0	0·0–673·0	7·9	28·2	2·0	0·0–16·5	0·0001
Bakery and pastry product stores	3·2	9·3	1·0	0·0–478·0	3·8	11·9	1·0	0·0–275·0	0·0038
Bakeries	8·4	28·6	2·0	0·0–144·0	3·9	15·4	1·0	0·0–204·0	< 0·0001
Dairy and cold cuts stores	0·9	3·8	0·0	0·0–51·0	0·3	1·5	0·0	0·0–22·0	< 0·0001
Ultra-processed foods	153·2	456·1	40·0	13·8–8301·0	61·7	237·1	13·0	4·4–124·8	0·0001
Bars	11·4	31·4	3·5	0·0–561·0	1·1	4·4	0·0	0·0–77·0	< 0·0001
Retail of beverages not consumed on the premises	11·4	28·7	3·0	0·0–458·0	4·1	16·2	1·0	0·0–299·0	< 0·0001
Convenience stores	0·3	1·6	0·0	0·0–27·0	0·7	4·0	0·0	0·0–83·0	< 0·0001
Candy and confectionery stores	3·5	15·8	0·0	0·0–308·0	1·6	8·6	0·0	0·0–169·0	< 0·0001
Snack bars	34·1	127·8	8·0	0·0–2483·0	14·4	73·9	2·0	0·0–1487·0	< 0·0001
Small grocery stores	68·1	169·0	18·0	0·0–2685·0	19·6	62·2	6·0	0·0–1180·0	< 0·0001
General or unspecified food product stores	11·5	48·0	2·0	0·0–912·0	6·1	27·8	1·0	0·0–545·0	< 0·0001
Prepared food outlets	1·2	7·7	0·0	0·0–159·0	3·0	18·0	0·0	0·0–288·0	< 0·0001
Bakery and pastry product stores	3·2	9·3	1·0	0·0–478·0	3·8	11·9	1·0	0·0–275·0	0·0038
Bakeries	8·4	28·6	2·0	0·0–144·0	3·9	15·4	1·0	0·0–204·0	< 0·0001
Supermarkets	7·6	17·2	3·0	0·0–268·0	7·0	15·4	3·0	0·0–240·0	0·0678
Hypermarkets	3·9	15·1	1·0	0·0–276·0	0·5	2·8	0·0	0·0–55·0	< 0·0001
Total	203·5	645·2	51·0	5·0–12093·0	93·3	388·6	18·0	1·0–7669·0	< 0·0001

* Wilcoxon test.

Regarding the density of food retail stores (Table 3), for the groups established according to the processing purpose, a reduction was observed for all food retail groups, with a reduction of 30·3 % in establishments providing fresh/minimally processed culinary ingredients, 25·7 % in establishments providing processed foods and 62·8 % in establishments providing ultra-processed foods. Despite presenting the greatest reduction, the group of establishments that source ultra-processed foods remained the group with the highest density among all groups. In addition, within the group of establishments that source ultra-processed foods, there was an increase in the density of bakeries (3·9 %) and the sale of prepared foods for consumption (5·5 %).

**Table 3:** Density of food retail establishments (per 10 000 inhabitants) by type in the municipalities of Rio Grande do Sul, Brazil, in 2010 and 2022

Type of establishment	2010				2022				*P*-value ^*^	Change	%
	Mean	sd	Median	Min.–max.	Mean	sd	Median	Min.–max.			
Fresh/minimally processed foods and culinary ingredients	22·8	13·6	20·6	0·0–111·5	14·0	8·7	13·1	0·0–95·8	< 0·0001	−1·92	–30·3
Butcher shops	3·5	3·5	2·8	0·0–21·3	1·0	1·6	0·0	0·0–9·6	< 0·0001	−2·52	–50·7
Fish markets	0·2	0·6	0·0	0·0–6·2	0·1	0·3	0·0	0·0–4·4	< 0·0001	−0·10	–12·1
Fruit and vegetable markets	1·6	2·5	0·6	0·0–25·6	1·0	1·8	0·0	0·0–16·5	< 0·0001	−0·55	–20·3
Canteens	0·1	0·4	0·0	0·0–4·9	0·1	0·3	0·0	0·0–4·7	< 0·0001	−0·06	–9·3
Restaurants	10·5	9·0	8·6	0·0–93·3	6·6	6·2	5·7	0·0–77·7	< 0·0001	−3·83	–24·6
Street vendors	0·5	1·0	0·0	0·0–6·3	0·2	0·6	0·0	0·0–7·2	< 0·0001	−3·84	–18·2
Supermarkets	4·9	4·2	4·3	0·0–38·9	4·9	4·2	4·0	0·0–35·1	0·5055	0·07	7·9
Hypermarkets	1·6	2·7	0·4	0·0–27·1	0·1	0·4	0·0	0·0–5·7	< 0·0001	−1·48	–47·2
Processed foods	5·2	3·8	4·7	0·0–21·4	3·3	2·7	3·2	0·0–16·5	< 0·0001	−1·87	–25·7
Bakery and pastry product stores	3·2	3·0	2·9	0·0–19·1	1·3	1·8	0·6	0·0–12·6	0·0119	−1·92	–43·1
Bakeries	1·7	2·3	1·0	0·0–19·1	1·9	2·3	1·4	0·0–16·5	< 0·0001	0·18	3·9
Dairy and cold cuts stores	0·2	0·6	0·0	0·0–6·7	0·9	0·5	0·0	0·0–8·5	< 0·0001	−0·13	–18·5
Ultra-processed foods	73·9	27·9	69·8	13·8–221·2	25·6	10·3	24·5	4·4–124·8	< 0·0001	−48·32	–62·8
Bars	7·1	8·0	5·0	0·0–67·0	0·4	0·8	0·0	0·0–7·4	< 0·0001	−6·78	–77·1
Retail of beverages not consumed on the premises	6·3	6·8	4·6	0·0–49·1	1·4	1·8	0·7	0·0–11·7	< 0·0001	−4·94	–55·5
Convenience stores	0·1	0·4	0·0	0·0–3·9	0·3	0·8	0·0	0·0–6·3	< 0·0001	−0·16	–0·9
Candy and confectionery stores	1·1	2·1	0·0	0·0–17·1	0·3	0·8	0·0	0·0–14·0	< 0·0001	−0·83	–25·6
Snack bars	15·0	10·9	13·4	0·0–71·1	3·6	3·7	3·1	0·0–23·2	< 0·0001	−11·32	–68·9
Small grocery stores	35·1	18·1	32·0	0·0–120·6	10·6	6·9	9·6	0·0–79·8	< 0·0001	−24·51	–66·2
General or unspecified food product stores	3·9	4·3	3·3	0·0–48·5	1·6	2·0	1·4	0·0–10·9	< 0·0001	−2·17	–50·3
Prepared food outlets	0·2	0·6	0·0	0·0–4·4	0·5	0·9	0·0	0·0–4·7	< 0·0001	0·26	5·5
Bakery and pastry product stores	3·2	3·0	2·9	4·6–0·0	1·3	1·8	0·6	0·0–12·6	0·0119	−1·92	–43·1
Bakeries	1·7	2·3	1·0	2·5–0·0	1·9	2·3	1·4	0·0–16·5	< 0·0001	0·18	3·9
Supermarkets	4·9	4·2	4·3	6·6–2·3	4·9	4·2	4·0	0·0–35·1	0·5055	0·07	7·9
Hypermarkets	1·6	2·7	0·4	2·1–0·0	0·1	0·4	0·0	0·0–5·7	< 0·0001	−1·48	–47·2
Total	96·7	36·8	91·7	0·0–27·1	36·5	19·2	34·2	0·0–5·7	< 0·0001	−60·3	–60·7

* Wilcoxon test.

Table 4 shows the food retail densities in relation to the sociodemographic variables of the municipalities. Municipalities that were wealthier, larger and with higher MHDI and lower illiteracy consistently had higher food retail densities, while municipalities with higher proportions of ethnic-racial minorities, higher illiteracy and lower income had lower densities of outlets sourcing healthy foods. In 2010, municipalities with high or very high MHDI values had a higher density of all food retail groups than municipalities with low or medium MHDI values did. Municipalities with a higher proportion of ethnic-racial minorities had a low density of the group of establishments sourcing fresh/minimally processed food and culinary ingredients. The municipalities with lower illiteracy rates had higher densities of all food retail establishments and of establishments in the groups providing fresh/minimally processed foods and culinary ingredients and processed foods. The municipalities in the highest income tertile had higher densities of all food retail establishments and of establishments in the fresh/minimally processed and culinary ingredients group, whereas compared with the municipalities in the other income tertiles, the municipalities in the second income tertile had the highest density of establishments in the ultra-processed group. Compared with municipalities of other sizes, municipalities with 20 000 to 50 000 inhabitants had a greater density of establishments in the fresh/minimally processed and culinary ingredients group, establishments in the processed group and establishments in the ultra-processed group. In 2022, similar patterns were observed in the associations for race, municipality size and illiteracy rate (Table 4).

**Table 4:** Density of food retail establishments in relation to sociodemographic variables of the municipalities of Rio Grande do Sul, Brazil, in 2010 and 2022

2010														
Variable	*n*	%	Total			Fresh/minimally processed foods and culinary ingredients			Processed foods			Ultra-processed foods		
			Median	Min.–max.	*P*-value	Median	Min.–max.	*P*-value	Median	Min.–max.	*P*-value	Median	Min.–max.	*P*-value
Municipality size*														
Small 1	396	79·8	88·6	13·8–268·7	0·0001^†^	19·7	0·0–111·5	0·0013^†^	4·3	0·0–21·4	0·0001^†^	67·5	13·8–221·2	0·0001^†^
Small 2	58	11·7	109·3	61·6–212·9		24·2	12·3–82·4		5·8	1·7–19·6		81·4	141·8–147·4	
Medium	24	4·8	98·9	69·5–161·7		22·3	12·6–38·5		5·6	3·4–11·3		77·1	17·3–120·3	
Large/metropolis	18	3·6	79·8	56·3–117·2		19·5	9·6–28·3		5·5	3·8–11·3		58·5	47·6–88·1	
MHDI														
Low/medium	176	35·5	84·8	13·8–209·1	0·0021^‡^	17·8	0·0–111·5	< 0·0001^‡^	3·8	0·0–19·6	< 0·0001^‡^	66·2	13·8–163·0	0·0481^‡^
High/very high	320	64·5	96·4	20·5–268·7		22·7	0·0–100·1		5·4	0·0–21·4		71·8	3·8–21·4	
Ethnic-racial minorities (%)^§,||^														
≤ 15 %	285	57·5	91·8	20·5–268·7	0·4184^‡^	21·6	0·0–111·5	0·0398^‡^	4·9	0·0–21·4	0·4476^‡^	69·8	16·3–221·2	0·5893^‡^
> 15 %	211	42·5	90·3	13·8–209·1		19·8	0·0–100·1		4·5	0·0–19·6		70·4	13·8–163·0	
Illiteracy rate^||^														
≤ 7 %	301	60·7	96·2	19·6–268·7	0·0087^‡^	22·3	0·0–111·5	< 0·0001^‡^	5·4	0·0–21·4	< 0·0001^‡^	71·8	16·3–221·2	0·0556^‡^
> 7 %	195	39·3	86·9	13·8–250·7		18·5	0·0–100·1		3·9	0·0–19·6		65·6	13·8–176·0	
Mean household income														
1st tertile	166	33·4	84·8	28·0–268·7	0·0002^†^	17·6	0·0–111·5	0·0001^†^	3·9	0·0–19·6	0·0001^†^	65·0	13·8–160·6	0·0013^†^
2nd tertile	165	33·3	100·6	28·0–268·7		21·2	0·0–91·7		5·1	0·0–21·4		75·5	22·8–189·9	
3st tertile	165	33·3	91·9	29·0–254·6		23·2	0·0–82·4		5·6	0·0–19·1		69·1	16·3–221·2	

2022														
Variable	*n*	%	Total			Fresh/minimally processed foods and culinary ingredients			Processed foods			Ultra-processed foods		
			Median	Min.–max.	*P*-value	Median	Min.–max.	*P*-value	Median	Min.–max.	*P*-value	Median	Min.–max.	*P*-value
Municipality size*														
Small 1	390	78·5	32·6	5·9–149·7	0·0001†	12·7	0·0–95·8		2·9	0·0–16·5		23·5	4·4–124·8	
Small 2	64	12·9	41	21·9–163·7		15·0	7·3–83·5	0·0029^†^	3·9	1·3–8·5	0·0015^†^	28·6	14·4–73·5	0·0001^†^
Medium	24	4·8	40·6	27·9–73·1		14·7	8·9–28·6		3·2	1·4–6·5		27·7	19·9–50·5	
Large/metropolis	19	3·8	42·2	23·1–59·0		14·9	6·4–22·8		3·8	1·9–5·7		27·6	17·9–36·3	
Ethnic-racial minorities (%)^§,||^														
≤ 20 %	297	60	34·4	5·9–163·7	0·6157^‡^	14·0	0·0–95·8	0·0053^‡^	3·3	0·0–16·5	0·7987^‡^	24·1	4·4–124·8	0·6102^‡^
> 20 %	200	40	34·7	5·6–81·5		12·5	0·0–37·6		3·2	0·0–15·0		25·0	6·4–57·6	
Illiteracy rate^||^														
≤ 5 %	314	63·2	36·9	5·9–163·7	< 0·0001^‡^	14·8	0·0–95·8	< 0·0001^‡^	3·6	0·0–16·5	< 0·0001^‡^	25·0	5·9–124·8	0·0115^‡^
> 5 %	183	36·8	31·4	6·1–63·3		10·8	0·0–37·6		2·3	0·0–10·3		23·5	4·4–48·9	

*Small 1: ≤ 20 000 inhabitants; small 2: > 20 000 to ≤ 50 000 inhabitants; medium: > 50 000 to ≤ 100 000 inhabitants; large/metropolis: > 100 000 inhabitants. ^†^Kruskal–Wallis test. ^‡^Mann–Whitney test. ^§^Black, Brown or Indigenous people were considered ethnic-racial minorities were considered ethnic-racial minorities. ^||^The 50th percentile of the variable distribution was used as the reference for categorisation.

**Fig. 1:**
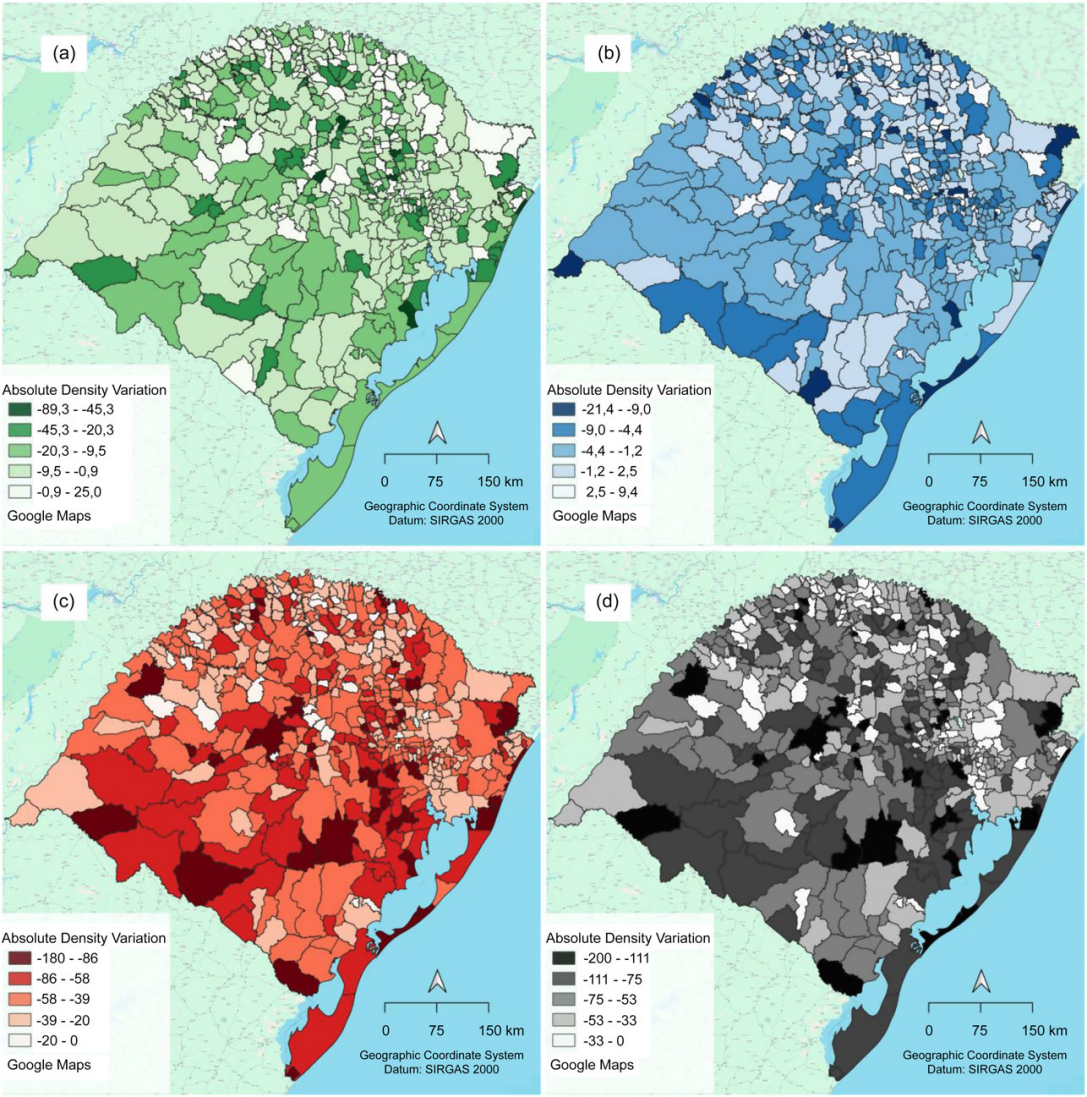
Absolute variation in the absolute density of total food retail establishments and by groups according to the level of processing of the predominantly sold foods in Rio Grande do Sul, Brazil, 2010–2022. (a) Fresh or minimally processed foods and culinary ingredients; (b) processed foods; (c) ultra-processed foods; (d) total.

The maps (Fig. 1) clearly illustrate the large reduction in absolute density of food retail establishments between 2010 and 2022. The largest reductions in all food retail groups are spread across the state but are observed predominantly in small municipalities. When the regions of the state were analysed, the south-central and western border regions were more affected by the reduction in the density of all food retail establishments and of each evaluated group than the other regions were. Moreover, compared with the other regions, the north-northeast region had the most municipalities with the least reduction in the density of all food retail establishments and of all groups evaluated.

In the analysis of the time trend of food retail density in RS from 2010 to 2022, a statistically significant reduction was observed for all groups of food establishments (Coef: –3·94; 95 % CI: –4·33, –3·54; *P* < 0·001), with a strong reduction in the ultra-processed group (Coef: –3·34; 95 % CI: –3·65, –3·02; *P* < 0·001) and a significant reduction in the group providing fresh/minimally processed foods and culinary ingredients (Coef: –0·50; 95 % CI: –0·67, –0·32; *P* < 0·001) and processed foods (Coef: –0·018; 95 % CI: –0·21, –0·15; *P* < 0·001) (online Supplementary Table 2). Despite the large reduction, the group of establishments providing ultra-processed products had the highest density among all groups, representing 59 % of the total establishments in 2022 (data not presented in tables). When the types of establishments that source ultra-processed foods were analysed separately, most presented a reduction in the analysed period, with the exceptions of establishments sourcing prepared foods for consumption (Coef: 0·07; 95 % CI: 0·05, 0·08; *P* < 0·001) and convenience stores (Coef: 0·01; 95 % CI: 0·01, 0·02; *P* = 0·010), which presented an increase, and bakeries, the only establishments that presented stable values (Coef: 0·02; 95 % CI: –0·02, 0·06; *P* = 0·284). Notably, mini-markets presented the greatest reduction among all establishments (Coef: 1·66; 95 % CI: –1·73, –1·59; *P* < 0·001; online Supplementary Table 2).

Municipalities with larger populations, higher income, higher MHDI, lower illiteracy and greater presence of ethnic-racial minorities experienced smaller reductions in the density of food retail establishments, both overall and across all groups of food sources (fresh/minimally processed, processed and ultra-processed). There was a smaller reduction in total food retail establishments (Coef: –2·97; 95 % CI: –3·34, –2·61; *P* < 0·001) and in all the groups providing fresh/minimally processed foods and culinary ingredients (Coef: –0·39; 95 % CI: –0·55, –0·24; *P* < 0·001), processed foods (Coef: –0·17; 95 % CI: –0·20, –0·14; *P* < 0·001) and ultra-processed foods (Coef: –2·56; 95 % CI: –2·83, –2·28; *P* < 0·001) in municipalities with populations greater than 100 000 (Coef: –2·97; 95 % CI: –3·34, –2·61) compared with the other municipalities. Compared with other municipalities, municipalities with high/very high MHDI values exhibited smaller reductions in the total density of establishments (Coef: –3·78; 95 % CI: –4·23, –3·33; *P* < 0·001), had a lower illiteracy rate (Coef: –3·77; 95 % CI: –4·21, –3·32; *P* < 0·001) and had a higher percentage of ethnic-racial minorities (Coef: –3·89; 95 % CI: –4·24, –3·54; *P* < 0·001). With respect to the income tertile, a dose–response effect was observed; the higher the income was, the smaller the reduction in the density of all types of establishments and of establishments in the fresh/minimally processed culinary ingredients group (Table 5).

**Table 5:** Temporal trend of food retail density in Rio Grande do Sul, Brazil (2010–2022), stratified by municipal sociodemographic variables

Variable	Total					Fresh/minimally processed foods and culinary ingredients					Processed foods					Ultra-processed foods				
	Mean	95 % CI	Coefficient	95 % CI	*P*-value^†^	Mean	95 % CI	Coefficient	95 % CI	*P*-value^†^	Mean	95 % CI	Coefficient	95 % CI	*P*-value^†^	Mean	95 % CI	Coefficient	95 % CI	*P*-value^†^
Municipality size*																				
Small 1	73·39	61·86, 84·93	−4·77	–5·13, –4·42	< 0·001	20·45	18·98, 21·91	−0·60	–0·77, –0·42	< 0·001	4·43	4·11, 4·75	−0·13	–0·16, –0·10	< 0·001	54·73	45·30, 64·16	−3·95	–4·28, –3·623	< 0·001
Small 2	85·79	74·76, 97·12	−4·62	–5·34, –3·91	< 0·001	24·32	22·94, 27·70	−0·55	–0·81, –0·29	< 0·001	5·63	5·15, 6·11	−0·20	–0·23, –0·16	< 0·001	61·94	52·32, 71·57	−3·98	–4·57, –3·39	< 0·001
Medium	75·16	63·92, 86·38	−4·68	–5·18, –4·19	< 0·001	19·41	18·04, 20·77	−0·52	–0·61, 0·42	< 0·001	4·69	4·18, 5·21	−0·21	–0·26, –0·16	< 0·001	55·47	46·04, 64·90	−3·96	–4·33, –3·58	< 0·001
Large/metropolis	65·83	58·78, 72·88	−2·97	–3·34, –2·61	< 0·001	19·97	19·03, 20·92	−0·39	–0·55, –0·24	< 0·001	4·89	4·48, 5·30	−0·17	–0·20, –0·14	< 0·001	45·77	39·72, 51·82	−2·56	–2·83, –2·28	< 0·001
MHDI																				
Low/medium	66·63	54·36, 78·90	−5·15	–5·46, –4·85	< 0·001	16·22	14·68, 17·75	−0·64	–0·72, –0·57	< 0·001	4·01	3·60, 4·42	−0·17	–0·20, –0·13	< 0·001	51·12	41·10, 61·14	−4·22	–4·50, –3·94	< 0·001
High/very high	73·07	64·04, 82·10	−3·78	–4·23, –3·33	< 0·001	21·35	20·20, 22·50	−0·48	–0·66, –0·29	< 0·001	5·01	4·59, 5·43	−0·18	–0·20, –0·15	< 0·001	52·04	44·39, 59·68	−3·22	3·57, –2·89	< 0·001
Ethnic-racial minorities (%)^‡, §^																				
≤ 15 %	75·04	65·34, 84·73	−4·00	–4·46, –3·54	< 0·001	21·68	20·49, 20·88	−0·45	–0·62, –0·27	< 0·001	4·92	4·54, 5·31	−0·16	–0·19, 0·14	< 0·001	54·02	45·87, 62·18	−3·41	–3·80, –3·02	< 0·001
> 15 %	70·21	60·98, 79·44	−3·89	–4·24, –3·54	< 0·001	19·98	18·78, 21·18	−0·52	–0·67, –0·37	< 0·001	4·85	4·41, 5·29	−0·18	–0·22, –0·15	< 0·001	50·37	42·58, 58·15	−3·28	–3·55, –3·02	< 0·001
Illiteracy rate^§^																				
≤ 7 %	78·92	63·91, 81·92	−3·77	–4·21, –3·32	< 0·001	21·34	20·19, 22·49	−0·48	–0·66, –0·29	< 0·001	5·03	4·60, 5·46	−0·18	–0·21, –0·15	< 0·001	51·94	44·29, 59·59	−3·22	–3·57, –2·87	< 0·001
> 7 %	68·48	56·59, 80·37	−4·93	–5·16, –4·71	< 0·001	17·07	15·60, 18·54	−0·61	–0·71, –0·51	< 0·001	4·03	3·67, 4·39	−0·14	–0·18, –0·10	< 0·001	51·84	42·19, 61·50	−4·04	–4·26, –3·83	< 0·001
Mean household income																				
1st tertile	63·89	52·44, 75·34	−4·79	–5·00, –4·58	< 0·001	15·89	14·42, 17·37	−0·62	–0·75, –0·50	< 0·001	4·04	3·67, 4·42	−0·15	–0·18, –0·12	< 0·001	49·17	39·77, 58·58	−3·96	–4·15, –3·77	< 0·001
2nd tertile	72·22	60·85, 83·59	−4·72	–5·03, –4·40	< 0·001	18·29	16·89, 19·69	−0·54	–0·61, –0·47	< 0·001	4·92	4·41, 5·43	−0·21	–0·24, –018	< 0·001	54·29	44·73, 63·84	−4·00	–4·28, –3·72	< 0·001
3st tertile	74·17	66·14, 82·20	−3·37	–3·92, –2·83	< 0·001	23·01	21·96, 24·07	−0·44	–0·67, –0·20	< 0·001	5·06	4·68, 5·45	−0·16	–0·19, –0·14	< 0·001	51·36	44·54, 58·17	−2·87	–3·27, –2·47	< 0·001
Total	72·27	68·85, 81·69	−3·94	–4·33, –3·54	< 0·001	20·71	19·52, 21·89	−0·50	–0·67, –0·32	< 0·001	4·88	4·47, 5·30	−0·18	–0·21, –0·15	< 0·001	51·93	43·99, 59·87	−3·34	–3·65, –3·02	< 0·001

MHDI, Municipal Human Development Index.

*Small 1: ≤ 20 000 inhabitants; small 2: > 20 000 to ≤ 50 000 inhabitants; medium: > 50 000 to ≤ 100 000 inhabitants; large/metropolis: > 100 000 inhabitants. ^†^Prais–Winsten regression. ^‡^Black, Brown or Indigenous people were considered ethnic-racial minorities. ^§^The 50th percentile of the variable distribution was used as the reference for categorisation.

In a comparison of the pre- and post-pandemic periods, there was a greater reduction in the food retail density of RS from 2020 to 2022 than from 2010 to 2019 for all groups of establishments, although the difference was NS (online Supplementary Table 2).

## Discussion

The results of this study reveal a significant reduction in establishments in all food retail groups in Rio Grande do Sul between 2010 and 2022 according to the analysis of the time trend. The period of this study (2010–2022) covers a period of economic recession in Brazil and RS^(32)^. The economic crisis has had a direct effect on food systems and altered the availability of food and the operation of establishments. During the recession, small and medium–sized merchants faced difficulties maintaining their business, and thus, food establishments could close or change ownership in a short time^(8,12,19)^. Rio Grande do Sul is composed mainly of small municipalities, and approximately 80 % of the municipalities have up to 20 000 inhabitants; generally, these municipalities also have small food retail stores, which are more susceptible to these scenarios. Although it was not possible to evaluate the size of the establishments in this study, the small municipalities had greater reductions in all types of food retail establishments than the large municipalities did during the analysed period. These results are similar to the findings of a study conducted in the Waterloo region, Ontario, Canada, which identified a decrease in access to healthy foods in small areas between 2011 and 2014^(15)^. Another study conducted in Mexico reported an increase in all retail and food products between 2010 and 2020, with the exception of small food establishments, which decreased^(12)^.

However, internationally, most studies that have evaluated food retail outlets over time have reported an increase in the number of food retail outlets^(8,9,12–14,18)^. In Brazil, two previous studies conducted in the city of Belo Horizonte, southeastern Brazil, evaluated changes in food retail establishments. Justiniano *et al*.^(11)^ evaluated the retail establishments in the municipality as a whole and observed an increase for all types of establishments between 2008 and 2018, with a greater increase for those with predominant sales of unhealthy foods (154·3 %). Freitas *et al*.^(19)^ evaluated food retail in the context of primary health care in the territory (2013–2018) and found stability in the total number of establishments in the analysed period, although when establishments were closed and new ones opened in the same place, they were not always of the same type and size. In areas with greater health vulnerability, several of the establishments selling fruits and vegetables closed and were not replaced. Notably, this study evaluated the dynamics of change in overall food retail at the state level, which may differ from the dynamics of individual municipalities, as the diversity can be much greater, according to the economic and demographic profile of each municipality.

The present study also evaluated the periods during and after the COVID-19 pandemic. The reduction in food retail outlets was even more pronounced in the post-pandemic period, which corroborates the international literature that reported an increase in the number of closed establishments^(16,17)^. This shows that the economic recession in addition to the health crisis had a significant negative effect on the food supply in the state of Rio Grande do Sul.

Among all reductions in food retail density in the state, the largest reduction concerned the group of establishments that sourced ultra-processed foods; however, even so, establishments in this group continued to be the most common among all groups. The density of this group was twice the density of retail stores that sourced fresh or minimally processed foods in 2022. In addition, ready-to-eat food establishments and convenience stores remained stable, whereas mini-markets were the establishments that presented the largest decrease during the period. Although they are classified as sources of ultra-processed foods in the methodology used, mini-markets could also be sources of fresh/minimally processed foods and culinary ingredients^(29)^. In this sense, the type of food actually sold in mini-markets varies greatly according to the municipality, and an onsite analysis is needed to understand the real impact of this reduction.

The significant reduction in the total number and density of mini-markets may be associated with the transformation of food systems in Latin America^(5)^, which is marked by a growing trend of consumption in supermarkets, to the detriment of small markets^(5,33–35)^. Likewise, in Brazil, POF data indicate that supermarkets are the main place where fresh, minimally processed and ultra-processed foods are purchased, which reflects the monopoly of large supermarket chains^(33–35)^. However, in these establishments, consumers are constantly induced to buy ultra-processed foods through pricing policies, the constant introduction of new products, promotions and marketing strategies promoting greater exposure of these foods on the shelves, among other strategies^(4)^. These large chains have also come to dominate digital food retail, which has been growing globally and tends to further intensify the promotion and sale of ultra-processed foods^(35)^. In the present study, although supermarkets experienced a reduction in food retail establishments, they were among the establishments with the smallest decreases in density over the analysed period.

Justiniano *et al*. reported a greater increase in establishments with predominant sales of ultra-processed foods than in other establishments, and among those with the greatest increase were convenience stores (157·5 %; *P* < 0·001) and delivery services (7088·5 %; *P* < 0·001)^(11)^. The increase in establishments with predominant sales of ultra-processed products is a global challenge, and its increase over the years has also been observed in municipalities in the USA^(9,13,20)^, Bangladesh^(14)^, Canada^(15)^ and Mexico^(12)^. Food retailing determines the ease with which people find food; thus, the increase in ultra-processed food establishments may impact their consumption, which increases weight gain and the incidence of Chronic Noncommunicable Diseases, such as diabetes mellitus^(4,5,7)^.

Regarding the social inequities in the availability of healthy foods observed in this study, in 2010, municipalities with low/medium MHDI values, high illiteracy rates and low average family income and in 2022, municipalities with high illiteracy rates had a significantly lower total density and number of establishments in the group providing fresh/minimally processed food and culinary ingredients than other municipalities. These results support the results of other Brazilian studies that evaluated food retail in municipalities^(36–40)^, which found that census tracts with low income have little diversity and a limited number of establishments^(36,41)^.

In the present study, changes in food retail also revealed patterns of social inequities: municipalities with lower illiteracy rates, larger populations, higher incomes and MHDI values presented a lower reduction in the density of all establishments and establishments that source fresh/minimally processed foods and culinary ingredients. This indicates that the reduction in food retail outlets disproportionately affected the most vulnerable municipalities; thus, sociodemographic inequalities remained throughout the years evaluated. Municipalities with low/medium MHDI values and high illiteracy rates and in the lowest income tertile had a greater reduction in the total number of establishments, establishments in the fresh/minimally processed culinary ingredients group and establishments in the ultra-processed group than other municipalities, which reflects a lower availability of all types of establishments in these municipalities. However, municipalities in the lowest income tertile had a greater reduction in all food retail establishments than other municipalities did, with the exception of establishments providing ultra-processed foods, which indicates a lower availability of healthy foods. These data are similar to those of Bilal *et al*., who evaluated the association between social and economic changes at the neighbourhood level from 2009 to 2013 and changes in food retail from 2013 to 2017 in Madrid (Spain) and reported that neighbourhoods with reduced socio-economic levels also presented a downward trend in the presence and proportion of establishments selling fruits and vegetables^(21)^.

The inequities observed in this study can also be observed in the maps. The south-central region of the state presents the greatest reduction in food retail establishments as a whole, and this region historically has lower income, education and health indices according to the socio-economic development index^(42)^. Moreover, in the map of the retail group providing fresh/minimally processed foods and culinary ingredients, a smaller reduction is highlighted in the north-northeast than in other regions, and it is the region with the highest income, education and health indices^(42)^.

With respect to possible ethnic-racial inequalities, municipalities with higher percentages of minorities had a smaller reduction in total food retail outlets than other municipalities did; however, they also showed a greater reduction in establishments in the fresh/minimally processed and culinary ingredients group and in the group providing processed foods, while there was a smaller reduction in the group providing ultra-processed foods. This reflects inequality in the availability of healthy foods for this population. Ethnic-racial inequalities over time have also been observed in studies in the USA: there was an increase in food retail outlets between 1990 and 2014, and regions with a greater proportion of ethnic-racial minorities presented a greater increase in establishments selling unhealthy food^(18)^. In New York, another study revealed low stability of establishments in areas with a predominance of ethnic-racial minorities between 2007 and 2011^(8)^. In Brazil, a study in Porto Alegre (RS) revealed that the census tracts with the highest proportion of ethnic-racial minorities were approximately twice as likely to be classified as food deserts, which indicates a direct relationship between social vulnerability and food availability^(41)^. These findings may represent the outcome of racism. In Brazil, although the history and experience of racism are different from those in the USA, where institutional racism predominates, structural racism persists. This is a deeply rooted system that perpetuates racial inequalities by shaping policies, institutions and living conditions in a way that privileges dominant groups and marginalises racialized populations, with negative impacts on their health and access to essential resources, such as food^(43,44)^.

Finally, monitoring possible changes in food retail in a context of crisis is essential. The economic crisis^(32)^, which was caused by COVID-19^(16,17)^, seems to have contributed significantly to the decrease in food retail establishments in the state of Rio Grande do Sul. In addition, in 2024, a climate emergency hit the state with the largest floods in its history^(45)^, which further aggravated this situation, as 15 000 food retailers were affected in some way by the floods, with healthy food retailers being the most affected proportionately^(46)^. The reduction in the supply of foods, especially healthy foods, combined with the higher retail density of establishments providing ultra-processed foods compared with establishments in other groups, poses a serious risk of food insecurity, especially among populations with high social vulnerability^(47)^.

### Limitations and strengths

This study has several limitations. The ecological approach does not permit drawing individual-level inferences from aggregated municipal data but generates hypotheses that must be tested by other study designs. Socio-economic data from the 2022 census were lacking, which prevented comparison with the 2010 census. The secondary data from the RAIS used to evaluate food retail in the state cover only the formal food market and do not include the informal market, indicators regarding food and nutrition safety equipment or digital food retail. However, this database includes a large number of food retailers and aligns with the broader mapping of the food environment in the country^(26)^. Finally, although the *Locais-Nova* classification was specifically developed for the Brazilian context, some misclassification regarding the purchasing profiles of establishments cannot be ruled out. Moreover, its application in other countries should be undertaken with caution. On the other hand, the study also has several strengths, such as its pioneering of the study of an entire Brazilian state; the analysis of various sociodemographic factors, including indicators and inequities in health; the use of a new classification system for food acquisition locations, in which establishments are classified as the sources of certain types of food according to the NOVA classification and the Food Guide for the Brazilian Population; and a decade-long analysis of food retailing throughout RS, including the period of the COVID-19 pandemic.

### Policy recommendations

On the basis of the results of this study, proposals can be developed to improve access to healthy foods throughout the national territory, with a focus on combating social and racial inequities. It is necessary to highlight the need for public policies that encourage the consumption of healthy foods through subsidies to establishments that source healthy foods, such as grocery stores and fairs, tax those providing unhealthy foods and regulate food retail to expand food access in vulnerable areas^(48)^. These measures should include targeted subsidies for small food retailers in socio-economically disadvantaged areas, urban planning strategies that limit the proliferation of ultra-processed food outlets and incentives for local food producers. Additionally, fiscal measures – such as taxation of ultra-processed foods and marketing restrictions – can support healthier consumer choices.

In addition to market-based strategies, strengthening public infrastructure for food and nutrition security can play a crucial role in addressing inequalities. In Brazil, this infrastructure is composed of a network of food security public facilities, which aim to ensure access to healthy food as a basic right. These include popular restaurants, community kitchens, public food markets, food banks, family farming supply centres and urban agriculture initiatives, often coordinated by municipal or state governments^(49)^. Caxias do Sul/RS offers a practical illustration of how public infrastructure can complement regulatory and fiscal policies. The municipality has institutionalised several public food security facilities, including a *Restaurante Popular* offering low-cost nutritious meals, four community kitchens serving around 1800 meals monthly, a food bank distributing over 100 tonnes of food each month and urban vegetable garden programmes linked to local agricultural production. These initiatives support immediate food access for vulnerable populations and reinforce sourcing from family farmers^(50)^. Finally, these interventions should explicitly address ethnic-racial disparities by incorporating race-conscious strategies to ensure equitable access to healthy foods and to counteract the structural racism embedded in food systems.

Future research should further explore the effects of food retail dynamics on dietary intake and health outcomes in vulnerable populations, particularly through studies with individual-level data and longitudinal designs. Mixed-methods approaches could help uncover the lived experiences of communities disproportionately affected by reductions in food availability.

## Supporting information

Schattschneider et al. supplementary material Schattschneider et al. supplementary material

## Supplementary material

For supplementary material accompanying this paper, visit https://doi.org/10.1017/S1368980025101286.
