# Supplementary material for: Social inequities in the community food environment: temporal analysis of food retail availability in the state of Rio Grande do Sul, Brazil (2010–2022)
Source: Public Health Nutr. 2025 Oct 14;28(1):e179. doi: 10.1017/S1368980025101286 (PMC12722071; doi:10.1017/S1368980025101286)
Supplement: Schattschneider et al. supplementary material [file S1368980025101286sup001.docx]

| **Supplementary Table 1 –** Changes in the density of food retail establishments in Rio Grande do Sul, Brazil, before and after the COVID-19 pandemic. | | | | | | | | | | | | |
| --- | --- | --- | --- | --- | --- | --- | --- | --- | --- | --- | --- | --- |
| **Period** | **Total** | | | **Fresh/minimally processed foods and culinary ingredients** | | | **Processed foods** | | | **Ultra-processed foods** | | |
|  | **Mean (95%CI)** | **β (95%CI)** | ***p*-value^a^** | **Mean (95%CI)** | **β (95%CI)** | ***p*-value^a^** | **Mean (95%CI)** | **β (95%CI)** | ***p*-value^a^** | **Mean (95%CI)** | **β (95%CI)** | ***p*-value^a^** |
| 2010 to 2019 | 78.4 (70.2;86.5) | -3.74 (-4.18;-3.31) | <0.001 | 21.5 (20.8;22.3) | -0.34 (-0.4;-0.26) | <0.001 | 5.2 (4.8;5.5) | -0.16 (-0.19;-0.13) | <0.001 | 57.0 (49.9;64.1) | -3.25 (-3.64; -2.86) | <0.001 |
| 2020 to 2022 | 52.0 (31.8;72.1) | -7.33 (-51.46; 36.80) | 0.281 | 17.9 (13.6;22.3) | -1.6 (-11.3;8.18) | 0.290 | 4.0 (3.2;4.7) | -0.27 (-1.73;1.20) | 0.261 | 35.1 (20.1;50.1) | -5.46 (-37.80;26.90) | 0.278 |
| **Total** | 72.3 (62.9;81.7) | -3.95 (-4.38;-3.52) | <0.001 | 20.7 (19.5;21.9) | -0.47 (-0.59;-0.34) | <0.001 | 4.9 (4.5;5.3) | -0.17 (-0.20;-0.15) | <0.001 | 51.9 (44.0;60.0) | -3.34 (-3.66;-3.02) | <0.001 |
| ^a^ Linear regression | | | | | | | | | | | | |

**Supplementary Table 2 -**Temporal trend of food retail density in Rio Grande do Sul, Brazil (2010–2022), by classification of food establishment

| **Type of establishment** | **Total of establishment** | | |
| --- | --- | --- | --- |
|  | **Mean (95%CI)** | **Coefficient (95%CI)** | ***p*-value^a^** |
| **Fresh/minimally processed foods and culinary ingredients** | 20.71 (19.52; 21.90) | -0.50 (-0.67; -0.32) | <0.001 |
| Butcher shops | 2.45 (2.10; 2.80) | -0.14 (-0.15; -0.13) | <0.001 |
| Fish markets | 0.21 (0.18; 0.23) | -0.01 (-0.01; -0.01) | <0.001 |
| Fruit and vegetable markets | 1.52 (1.39; 1.64) | -0.05 (-0.02; -0.04) | <0.001 |
| Canteens | 0.19 (0.16; 0.22) | -0.01 (-0.02; 0.01) | 0.172 |
| Restaurants | 11.27 (10.92; 11.61) | -0.12 (-0.26; 0.02) | 0.084 |
| Street vendors | 0.65 (0.57; 0.74) | -0.04 (-0.06; -0.01) | <0.001 |
| Supermarkets | 3.48 (3.41; 3.55) | -0.02 (-0.03; -0.01) | 0.009 |
| Hypermarkets | 0.95 (0.68; 1.22) | -0.12 (-0.14; -0.10) | <0.001 |
| **Processed foods** | 4.89 (4.47; 5.30) | -0.18 (-0.21; -0.15) | <0.001 |
| Bakery and pastry product stores | 3.20 (2.78; 3.62) | -0.17 (-0.24; -0.11) | <0.001 |
| Bakeries | 1.37 (1.30; 1.45) | -0.02 (-0.02; 0.06) | 0.268 |
| Dairy and cold cuts stores | 0.31 (0.25; 0.36) | -0.02 (-0.03; -0.02) | <0.001 |
| **Ultra-processed foods** | 51.93 (43.99; 59.86) | -3.33 (-3.66; -3.02) | <0.001 |
| Bars | 2.95 (1.63; 4.26) | -0.48 (-0.68; -0.28) | <0.001 |
| Retail of beverages not consumed on the premises | 3.75 (3.14; 4.36) | -0.25 (-0.28; -0.23) | <0.001 |
| Convenience stores | 0.20 (0.19; 0.24) | 0.01 (0.00; 0.02) | 0.009 |
| Candy and confectionery stores | 1.19 (1.03; 1.35) | -0.07 (-0.70; -0.06) | <0.001 |
| Snack bars | 11.83 (10.21; 13.46) | -0.68 (-0.75; -0.61) | <0.001 |
| Small grocery stores | 21.51 (17.44; 25.57) | -1.66 (-1.73; -1.59) | <0.001 |
| General or unspecified food product stores | 4.27 (3.88; 4.66) | -0.15 (-0.17; -0.13) | <0.001 |
| Prepared food outlets | 0.97 (0.81; 1.14) | 0.07 (0.05; 0.08) | <0.001 |
| Bakery and pastry product stores | 3.20 (2.78; 3.62) | -0.17 (-0.24; -0.11) | <0.001 |
| Bakeries | 1.37 (1.30; 1.45) | -0.02 (-0.02; 0.06) | 0.268 |
| Supermarkets | 3.48 (3.41; 3.55) | -0.02 (-0.03; -0.01) | 0.009 |
| Hypermarkets | 0.95 (0.68; 1.22) | -0.12 (-0.14; -0.10) | <0.001 |
| **Total** | 72,27 (68,85; 81,69) | -3,94 (-4,33; -3,54) | <0,001 |

a- Prais-Winsten regression
